# Pilot Efficacy of a DriveFocus™ Intervention on the Driving Performance of Young Drivers

**DOI:** 10.3389/fpubh.2018.00125

**Published:** 2018-05-04

**Authors:** Liliana Alvarez, Sherrilene Classen, Shabnam Medhizadah, Melissa Knott, Wenqing He

**Affiliations:** ^1^School of Occupational Therapy, Faculty of Health Sciences, University of Western Ontario, London, ON, Canada; ^2^Department of Occupational Therapy, College of Public Health and Health Professions, University of Florida, Gainesville, FL, United States; ^3^Department of Statistical and Actuarial Sciences, Faculty of Science, University of Western Ontario, London, ON, Canada

**Keywords:** youth, driving, hazard anticipation, efficacy, novice drivers

## Abstract

Road traffic injuries are the leading cause of death for youth between the ages of 15 and 29 around the world. A need remains for evidence-based interventions that can improve the underlying skills of young drivers, including hazard perception and anticipation. This pilot study investigated the preliminary impact of a six session DriveFocus™ intervention on the ability of young novice drivers (mean age = 18.6, SD = 2.12) to detect (visual scanning), and respond (adjustment to stimuli) to critical roadway information. Using a CDS-200 DriveSafety™ simulator, drives were recorded and sent to a blinded evaluator (occupational therapist), who scored the recorded drives for number and type (visual scanning and adjustment to stimuli) of errors. We observed a statistically significant decline in the number of visual scanning [*t*(34) = 2.853, *p* = 0.007], adjustment to stimuli [*t*(34) = 3.481, *p* = 0.001], and total driving errors [*t*(34) = 3.481, *p* = 0.002], among baseline and post-test 2.

## Introduction

Road traffic injuries are the only accidental cause of death in the World Health Organization’s top 10 list. Moreover, in spite of being highly preventable, road traffic injuries remain the number one killer of youth between the ages of 15 and 29 (1). In the United States alone, one-third of all youth deaths are attributed to road traffic injuries (2). The overrepresentation of youth in road traffic injuries and fatalities has been attributed to multiple factors including: brain maturation and risk-taking behaviors (3, 4), inexperience (5), driving under the influence of alcohol or drugs (6), and distracted driving and hazard perception (7). National and local government agencies around the world have led efforts to curtail the impact of this public health threat through law enforcement, graduated licensing, and awareness among road users (1). Although such strategies represent important progress, road traffic injuries among youth continue to be dangerously high. Therefore, a need remains for efficacious and effective intervention strategies that can improve the driving skills of young drivers.

A critical antecedent of youth’s driving risk has distracted driving. Although distraction poses a risk for divers of all ages, drivers under the age of 20 have the highest fatality rates attributed to distraction (8). Distracted driving can occur when a driver takes their eyes off the road (visual distraction), their hands off the wheel (manual distraction), or their mind off the driving task (cognitive distraction) (8). Not surprisingly, texting and driving has been the focus of numerous educational and law enforcement campaigns as operating a phone involves all three types of distraction. However, a growing body of literature reveals that in the absence of a phone, young novice drivers are still at a high risk for crashes as a result of hazard anticipation errors (9–11).

Hazard anticipation, also known as hazard perception, comprises “a set of driver behaviors including: (1) the awareness and knowledge of roadway risks and associated threats to driving safety; (2) visual search that facilitates detection and recognition of elements directly or indirectly contributing to unsafe situations; (3) prediction of emerging and latent hazards based on information from the visual scene; and (4) execution of driving responses to avoid or minimize potential conflicts due to recognized hazards” [(11), p. S16]. When compared with experienced adult drivers, young novice drivers show poorer hazard anticipation (12), are less likely to visually scan locations where hazards are likely to emerge (13), and are less likely to demonstrate speed control and adequate vehicle positioning to avoid a hazard (14).

Traditional driver’s education classes fail to show significant improvement of hazard anticipation skills for this population (15, 16). In addition, computer or multimedia-based training has yielded superior results over paper-based training (17). Several computer or multimedia-based hazard anticipation programs have been developed for young drivers [for a review see Ref. (11, 18)]. Based on McDonald and colleagues’ classification, the multimedia-based approaches documented in the literature include interactive computer-based approaches and video-based approaches.

Interactive computer-based approaches such as the well-studied risk awareness and perception training (RAPT) program (13), use a PC-based training to show the driver snapshots of driving scenarios. In the RAPT program, the driver is then asked to use the mouse to click on areas of the snapshot that they believe would warrant special attention if he/she was driving through that scenario. After watching the snapshots of each scenario, drivers receive an explanation of the hazard that is present, and a top down display is used to further illustrate the identified hazard (19). Available in different versions and with updated content between versions, RAPT has been evaluated in both on-road and driving simulator studies with promising results. Specifically, the RAPT program increases the percentage of correct glances upon predefined locations where there is a potential risk. The RAPT program provides a successful in-laboratory approach to hazard anticipation training. However, given the nature of the driving task, research has identified the need to augment this training by including opportunities for multiple glances at areas of interest (vs. one critical area per snapshot) (18). A static snapshot display allows only a few seconds for the driver to detect and react to a potential hazard. Thus, using a dynamic display would allow on-going driver interaction and provide a closer approximation of real-life driving situations. In addition, being an in-laboratory intervention, the RAPT program may pose acceptability, access and usability challenges for the young population. Thus, this study integrated hazard perception training into a dynamic display to determine the preliminary impact of such interventions on the underlying skills of young drivers.

Video-based approaches, also known as commentary driving, involve the use of videos to elicit verbal descriptions of the scenes, a description of what the driver is thinking and planning, or a response to a series of questions pertaining the driver’s assessment of the situation and the appropriate responses (20). Studies evaluating such approaches have reported promising improvement in reaction times during post-test performance of hazard perception dual tasks or a video-based speed tests (21, 22). However, the consistent transfer of such outcomes to drive performance or fitness to drive remains poorly understood as researchers did not include simulator or on-road assessments.

In summary, available intervention strategies capitalize on the use of computer or multimedia approaches with positive outcomes. However, the suitability of the interventions remains unclear as the available interactive computer-based approach uses static displays that do not resemble the dynamic nature of on-road hazard anticipation, or require in-laboratory training. In addition, video-based approaches have not been used to assess driving performance on a driving simulator, or on-road fitness to drive abilities of young novice drivers. Thus, a twofold opportunity arises: to leverage the use of a dynamic interactive multimedia-based intervention; and assessing the corresponding ability of teens to detect (visual scanning) and respond (adjustment to stimuli) to critical roadway information, *via* a driving simulator. Thus, this study investigated the preliminary efficacy of an intervention that integrates a dynamic interactive display and real-life footage of drive, since a first-person view to determine the preliminary impact of such interventions on the underlying skills of young drivers.

Given teens’ wide adoption of technology, this pilot study investigated the efficacy of the DriveFocus™ app as an intervention on the ability of teens to detect (visual scanning) and respond (adjustment to stimuli) to critical roadway information. Thus, our primary outcomes measures included the number of visual scanning, adjustment to stimuli, and total errors made by young novice drivers, assessed *via* a DriveSafety™ CDS-200 high fidelity driving simulator. The fidelity and usability tested DriveFocus™ app (23), was developed by an occupational therapist, who was also a certified driver rehabilitation specialist. The app utilizes a dynamic display consisting of real drives in a variety of cities across North America (a total of five available at the time of the study: Ontario, Quebec, Florida, South Carolina, and Vermont) and instructs the user on how to identify and prioritize critical roadway information. We hypothesized that the total number of driving errors, visual scanning, and adjustment to stimuli errors made by young novice drivers in response to the DriveFocus™ intervention would differ among baseline, post-test 1 and 2.

## Materials and Methods

This study was approved by the University of Western Ontario, Non-medical Research Ethics Board (#107267). The study was also registered as an intervention trial in ISRCTN (study ID: ISRCTN66950576). All participants provided informed consent prior to enrolling in the study. For participants under 18 years of age, the parent or legal guardian provided informed consent. Participants received a $25 movie theater gift card and a $20 gas voucher for their participation in the study.

### Design

We conducted a pilot, single arm repeated measures (pretest and 2 post-tests) study (*N* = 39), powered to detect an effect of *f* = 0.4. with a two-tailed α = 0.05, β = 70%. Current recommendations in the extant literature include a sample size of 10% the number required for a larger trial (24), while others recommend 15 participants per arm for medium effects (25). Thus, our calculations ensured that we are above those pilot study recommendations, while providing objective metrics for the achieved number.

### Participants

Participants were recruited *via* flyers and posters around the university campus, as well as local shopping malls, movie theaters, and youth community organizations. The research team presented the study opportunity at local driving schools. Ads were distributed through social media and pre-movie advertisements in a local movie theater.

Participants were included if they: (1) were between 16 and 22 years of age; (2) had a valid G1 or G2 driver’s license (in Ontario, Canada, such licenses are equivalent to a learner’s permit allowing the driver to drive only with an accompanying fully licensed driver in the vehicle (G1), or with restrictions in the number of passengers and the time of day (G2)); (3) were able to read and comprehend English as per self or parental report; and (4) were able to travel to the research laboratory for the duration of the study.

Participants were excluded from the study if they: (1) had been diagnosed with a mental health or neurological condition; (2) were taking medications that precluded participation as a result of side effects; (3) did not meet the ministry of transportation of Ontario (MTO)’s visual acuity requirements (20/50 in both eyes examined together) as per in-lab assessment; and (4) did not meet the MTO’s peripheral field of view (120° horizontal field) as per in-lab assessment.

### Measures

Using a standardized intake form (26) we obtained demographic information including: age, gender, ethnicity, and education level. Participants completed an adapted driving history from Ali et al. (27). Section one of this questionnaire obtained information regarding participants’ type of license, type of driver’s education received, collision involvement, and citations. Section two asked participants to rate in a scale from “never” to “always” on perceiving themselves as engaging in 11 driving habits (e.g., making or answering a phone call while driving). In addition, participants were asked to keep a journal of their driving throughout the study, reporting the length (in time) of their drives, frequency of their drives per week (number), type of drive (e.g., practice driving, personal, etc.), and level of supervision (e.g., driving school instructor, fully licensed driver, etc.).

Participants also completed an evidence-based battery of cognitive, motor, and visual assessments appropriate for this population (26). Cognitive assessments included the comprehensive trail-making test (28) for mental flexibility and set-shifting; and the SDMT [Symbol Digit Modalities Test; (29)] for simple and complex sequencing. Motor proficiency was assessed *via* the BOT-2 [Bruininks–Oseretsky Test of Motor Proficiency; (30)], while visual motor integration was assessed via the Beery VMI™ [Beery-Buktenica Developmental Test of Visual-Motor Integration; (31)]. The Optec 2500^®^ Visual Analyzer (Stereo Optical Company, Inc., Chicago, IL, USA) was used to assess eight visual skills, including visual acuity, peripheral visual fields, contrast sensitivity, depth perception, color discrimination, and lateral and vertical phorias. The results of the clinical assessments are not further discussed in this manuscript.

Two members of the research team administered the simulator assessment. All drives were recorded from the dashboard view to capture the driver’s eye gaze, as well as from the driver’s viewpoint. Once a participant completed the protocol, the set of drives were sent to an evaluator (occupational therapist), who remained blinded to the status (pre or post-test) of the drive. The trained evaluator used a scoring sheet adapted for this study, to assess visual scanning (appropriate movement and turning of the eyes, neck, and head to gaze at oncoming objects and roadway information); and adjustment to stimuli (driver’s response to driving situations including adjusting the speed in response to a traffic light, or breaking in response to the emergence of a pedestrian on a roadway). Participants were also screened for symptoms of simulator sickness before and after each drive using the modified version (32) of the motion sickness assessment questionnaire (MSAQ) (33). The modified MSAQ asks the participant to rate whether they are experiencing four symptoms on a scale from 0 (not at all) to 10 (severely). The symptoms include queasiness, dizziness, sweatiness, and nausea.

### Intervention

**Figure 1:**
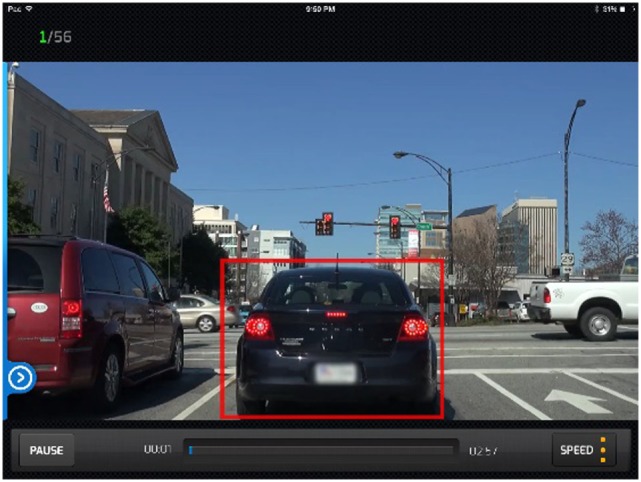
DriveFocus™ interactive drive requires participants to touch the lights of the vehicle in front as well as the traffic lights. The lights of the leading vehicle should be prioritized.

The DriveFocus™ intervention is an interactive app (designed to run in a tablet format) that teaches the driver to identify critical items on the road and prioritize them in terms of their potential hazard and necessary action. The critical items include 11 categories: stop signs, traffic lights, yield signs, braking lights and turning signals of the lead vehicle, pedestrians and bicyclists, regulatory signs, caution signs, pavement markings, vehicles entering from the left or right, construction signs, and objects in the driver’s path. During interactive video drives containing real footage of drives throughout North America (referred to as tours), participants must touch on the critical item on the screen as soon as they identify it on the interactive video tour. Also, participants must touch on multiple critical objects in the video in adequate order, prioritizing them as instructed. If touched correctly, the item is surrounded by a red square (see Figure 1) and participants hear a tone that provides positive feedback or a buzz indicating the object was misidentified. For example, when approaching an intersection with a green light while the car in front has its brake lights on (both critical items); the brake lights take priority over the green light because the driver may need to slow down or stop even before arriving to the light. In this case, the user is instructed to touch the brake lights before the green light, as seen in Figure 1.

Each tour consists of six interactive video drives. The drives are ordered in increasing complexity and are 3–5 min in duration. The most difficult drives have more critical items. After a drive is completed the user can see his/her scores for the drive. The app will score the participant on percentage of critical items noticed, percentage of time that the items were noticed in proper sequence (e.g., the brake lights on the car directly in front of them take precedence over a green light), and reaction times (measured in seconds). Throughout the drives, the app provides feedback to the participants *via* an easy-to-understand graphical representation of the drive that indicated how well they performed, their scores, and any missed objects throughout the video. Participants unlocked different levels of complexity based on their scores, to motivate their interaction throughout the session.

The intervention was administered in six sessions, lasting approximately 45 min each and occurring over a 9-week period. On the first session, one of the researchers or research assistants provided participants with a guided overview and description of each of the app’s sections. Participants then completed the app’s training section (including six practice interactive video drives). The training section includes an overview of the app, a review of the 11 categories of critical items, the best practices to prioritize them, and the scoring. In subsequent sessions participants independently moved along the full series of tours (one per session).

### Procedure

After enrolling into the study participants completed the intake form and the clinical battery of assessments. For participants under 18 years of age, the parent or legal guardian completed the intake form. Participants then completed the baseline simulator assessment.

The simulator assessment consisted of a 7-min acclimation drive to mitigate potential onset of simulator sickness (34, 35). The acclimation drive was followed by one of three possible 15-min test drives that were programmed for random assignment. The acclimation drive was part of an evidence-based simulator sickness mitigation protocol. While allowing participants to become comfortable with the simulator, the acclimation drive progressively introduced more complex graphics and maneuvers (e.g., starting with a straight drive with no traffic progressing toward a residential area practicing making right turns). The test drives started in a residential area and moved to an urban/downtown area. All test drives included five scripted hazardous events, programmed to challenge participants’ visual scanning and adjustment to stimuli abilities. These scripted events included: unexpected pedestrian crossing; car making a rapid lane change in front of the driver; sudden change in traffic lights (go-no-go); a way-finding task; and a car suddenly pulling out of a driveway. Order and location of the scripted events was pseudo-randomized across the three drives (i.e., order and location was randomly chosen and then programmed into the scenarios, and each scenario consistently presented its pre-programmed order of events).

After the baseline assessment, participants completed three 1-h intervention sessions (1/week × 3 weeks), followed by post-test 1 (simulator assessment only); three 1-h intervention sessions; and post-test 2 (clinical and simulator assessment).

### Analysis

All data were entered into a password protected SPSS (v. 24, IBM, New York, U.S.) database. A member of the research team performed checks to ensure accuracy of data entry. We used descriptive statistics to summarize all variables (i.e., frequencies, percentages, means, and SDs). We then conducted related-samples *t*-test among the three time points (baseline, post-test 1, and post-test 2) to detect statistically significant differences (*p* < 0.05) in the primary outcome variables: number of visual scanning, adjustment to stimuli, and total number of driving errors made by the participants.

## Results

A total of 39 participants were enrolled in the study (mean age = 18.6 years old ±2.12; 71.8% females). Four participants did not complete all the sessions of the protocol, and were thus excluded from further analysis. Table 1 shows the descriptive statistics for the sample.

**Table 1:** Descriptive statistics for demographic, driving history, and practice journal variables (*n* = 39).

Variable	Statistic
Demographic variables	
Age, M(SD)	18.61 (2.1)
Gender, *n* (%)	
Male	11 (28.2)
Female	28 (71.8)
Ethnicity, *n* (%)	
Caucasian	22 (56.4)
Hispanic	2 (5.1)
Asian	13 (33.3)
African	2 (5.1)
Occupation, *n* (%)	
Grade 10 student	1 (2.6)
Grade 11 student	11 (28.2)
Grade 12 student	2 (5.1)
College/university student	22 (56.4)
Full/part time employ	1 (2.6)
None of the above	2 (5.1)

Overall, participants were mostly female, Caucasian, and college or university students. Table 2 reports the descriptive statistics for the driving history and habits questionnaire and the practice journal. Most participants (59%) had a G1 license, meaning they had approved the theoretical examination and were able to drive with an experienced licensed driver in the passenger seat. Most participants reported having received formal driver’s education, with an MTO approved course, with classroom training and an on-road component. Most participants had not been involved in crashes, nor had they received citations or traffic tickets. Regarding their driving habits, most participants admitted to: occasionally exceed the speed limit, occasionally listen to or adjust a media player, frequently talk with passengers, and occasionally eat or drink while driving. For all other driving habits, most participants indicated never to engage in those behaviors.

**Table 2:** Descriptive statistics for self-reported driving history and habits questionnaire and practice journal variables (*N* = 39).

**Driving history variables**	
Type of license, *n* (%)	
G1	23 (59.0)
G2	16 (41.0)
Have you received formal driver’s education, *n* (%)	
Yes	24 (61.5)
No	15 (38.5)
Type of driver’s education received, *n* (%)	
None	15 (38.5)
MTO-approved driving course	19 (48.7)
Classroom training	20 (51.3)
On-road training	19 (48.7)
Computer-based training	3 (7.7)
Simulator training	1 (2.6)
Instruction from family member	15 (38.5)
Number of crashes you have been involved in, *n* (%)	
0	36 (92.3)
1	1 (2.6)
Missing/prefer not to answer	2 (5.2)
Number of citations or traffic tickets, *n* (%)	
0	35 (89.7)
1	1 (2.6)
Missing/prefer not to answer	3 (7.7)

**Driving habits variables**	
Exceeding the speed limit, *n* (%)	
Never	4 (10.3)
Hardly ever	7 (17.9)
Occasionally	14 (35.9)
Quite often	4 (10.3)
Frequently	5 (12.8)
Nearly all the time	0
Missing/prefer not to answer	5 (12.8)
Racing with neighboring cars, *n* (%)	
Never	24 (61.5)
Hardly ever	10 (25.6)
Missing/prefer not to answer	5 (12.8)
Driving in the form of sutures, *n* (%)	
Never	16 (41.0)
Hardly ever	9 (23.1)
Occasionally	8 (20.5)
Quite often	1 (2.6)
Missing/prefer not to answer	5 (12.8)
Using the seatbelts, *n* (%)	
Nearly all the time	34 (87.2)
Missing/prefer not to answer	5 (12.8)
Making or answering a call while driving, *n* (%)	
Never	27 (69.2)
Hardly ever	3 (7.7)
Occasionally	1 (2.6)
Quite often	3 (7.7)
Missing/prefer not to answer	5 (12.8)
Reading or receiving text messages while driving, *n* (%)	
Never	22 (56.4)
Hardly ever	8 (20.5)
Occasionally	2 (5.1)
Quite often	1 (2.6)
Missing/prefer not to answer	5 (12.8)
Listening to or adjusting a media player while driving, *n* (%)	
Never	1 (2.6)
Hardly ever	4 (10.3)
Occasionally	8 (20.5)
Quite often	7 (17.9)
Frequently	6 (15.4)
Nearly all the time	7 (17.9)
Missing/prefer not to answer	6 (15.4)
Watching a display screen while driving, *n* (%)	
Never	13 (33.3)
Hardly ever	10 (25.6)
Occasionally	5 (12.8)
Quite often	3 (7.7)
Frequently	2 (5.1)
Missing/prefer not to answer	6 (15.4)
Talking with passengers while driving, *n* (%)	
Never	1 (2.6)
Occasionally	3 (7.7)
Quite often	8 (20.5)
Frequently	16 (41.0)
Nearly all the time	5 (12.8)
Missing/prefer not to answer	6 (15.4)
Eating or drinking while driving, *n* (%)	
Never	10 (25.6)
Hardly ever	8 (20.5)
Occasionally	11 (28.2)
Quite often	4 (10.3)
Missing/prefer not to answer	6 (15.4)

**Practice journal variables**	
Driving time, *n* (%)	
None	36 (92.3)
2–4 h/week	1 (2.6)
4–6 h/week	1 (2.6)
More than 6 h/week	1 (2.6)
Driving practice type, *n* (%)	
None	36 (92.3)
Personal	3 (7.7)
Supervision while driving, *n* (%)	
No practice	36 (92.3)
Unsupervised	3 (7.7)

*MTO = ministry of transportation of Ontario; only response options for which there was at least one responded are included in the table for each variable; frequencies and percentages for type of driver’s education total more than 100% as participants could have received more than one type of training. G1 = first stage of graduated licensing program. Driver must be accompanied by a fully licensed driver at all times; G2 = second stage of graduated licensing program. Driver can drive independently. If the driver is under 19 years of age and is driving between midnight and 05:00 a.m., they can only drive with one passenger under the age of 19 in the vehicle*.

Table 3 presents the descriptive statistics for the primary outcomes, per type and number of driving errors, at baseline, post-test 1 and post-test 2.

**Table 3:** Type and number of errors among baseline, post-test 1, and post-test 2 (*N* = 34).

Variable	Baseline mean (SD)	Post-test 1 mean (SD)	Post-test 2 mean (SD)
Number of visual scanning errors	34.06 (14.58)	29.26 (14.58)	27.06 (8.52)

Number of adjustment to stimuli errors	19.17 (11.99)	15.60 (9.41)	12.28 (5.70)

Total number errors	53.23 (25.21)	44.83 (23.04)	39.40 (13.36)

The paired-sample *t*-tests for each variable among baseline, post-test 1, and post-test 2, indicated that the DriveFocus™ intervention elicited statistically significant decrease in: the number of visual scanning of 7.000 (95% CI, 2.013–11.986) errors, *t*(34) = 2.853, *p* = 0.007 between baseline and post-test 2; the number of adjustment to stimuli of 6.885 (95% CI, 2.866–10.905) errors, *t*(34) = 3.481, *p* = 0.001 between baseline and post-test 2; the number of adjustment to stimuli of 3.314 (95% CI, 0.065–6.563) errors, *t*(34) = 2.073, *p* = 0.046 between post-test 1 and post-test 2; and finally, the total number of 13.829 (95% CI, 5.380–22.278) errors, *t*(34) = 3.481, *p* = 0.002 between baseline and post-test 2.

**Figure 2:**
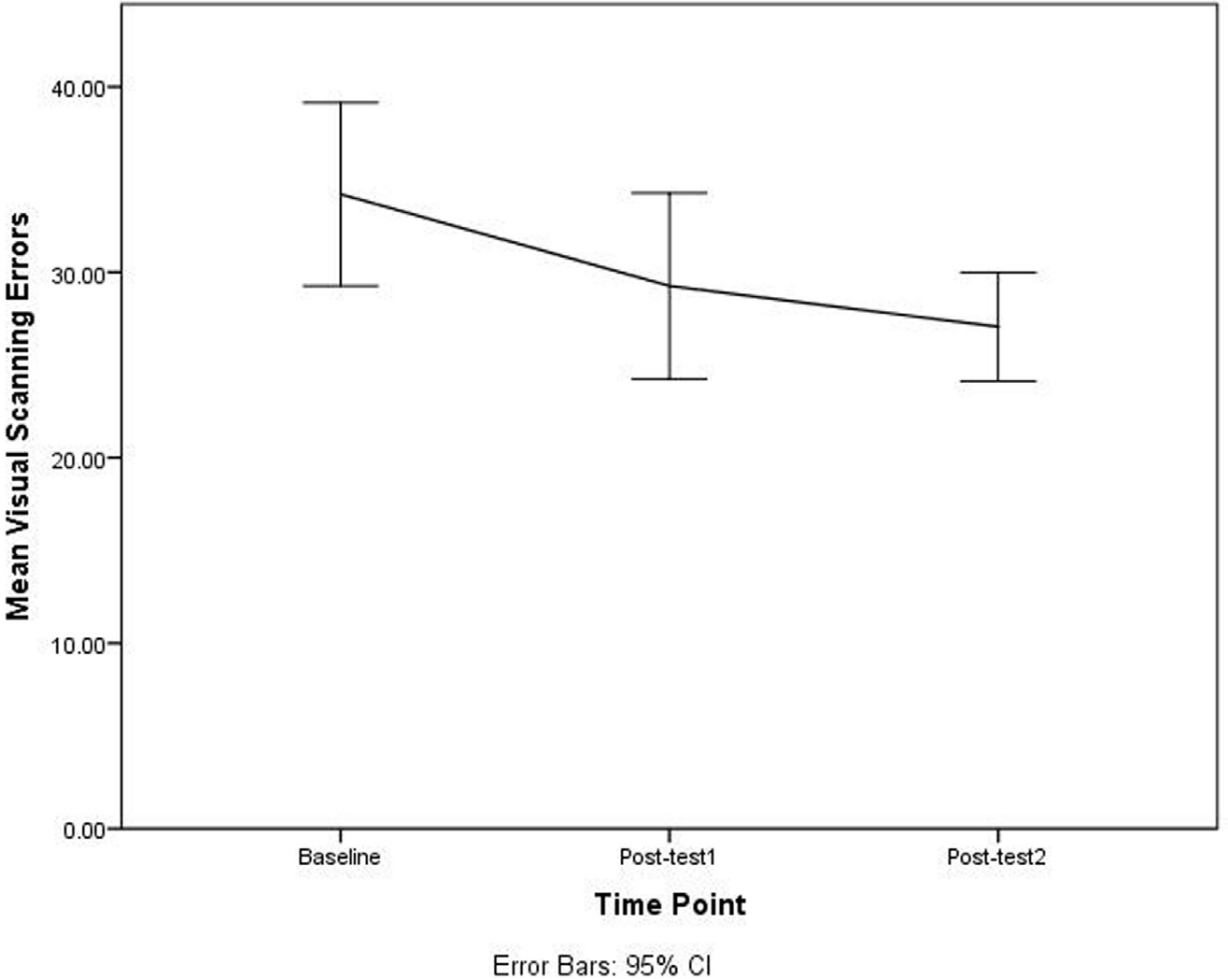
Number of visual scanning errors among baseline, post-test 1, and post-test 2.

**Figure 3:**
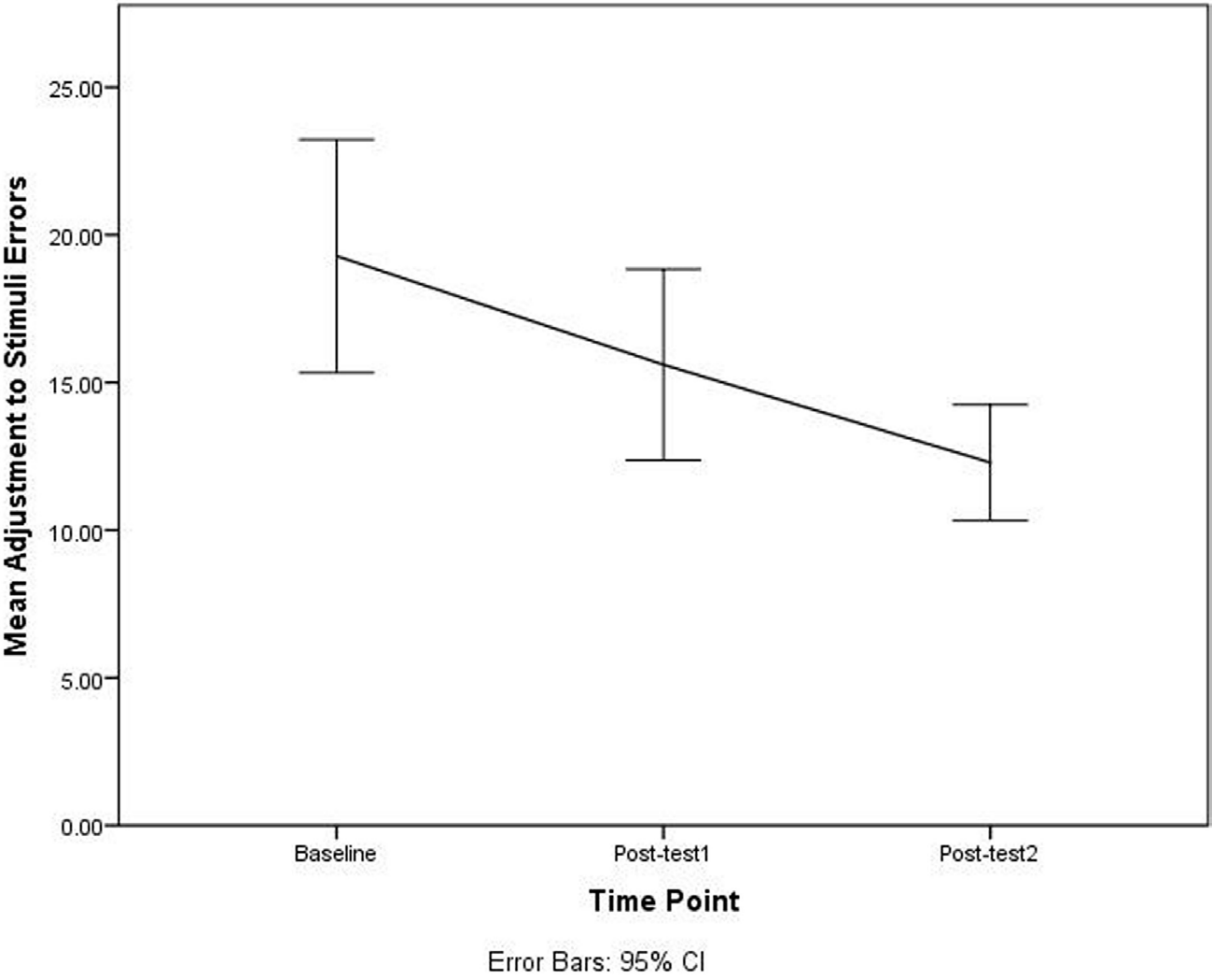
Number of adjustment to stimuli errors among baseline, post-test 1, and post-test 2.

**Figure 4:**
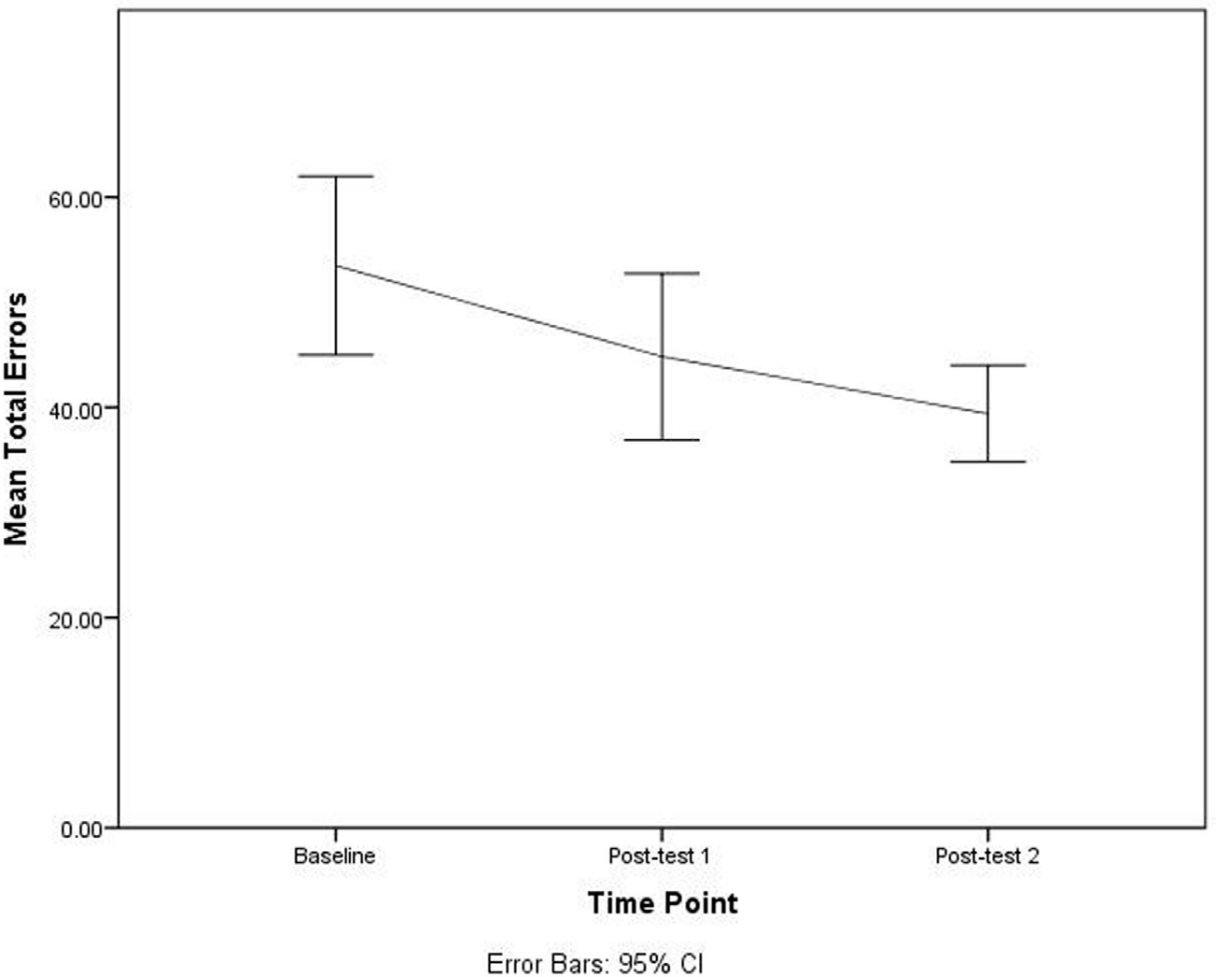
Total number of errors among baseline, post-test 1, and post-test 2.

Although there was a decrease in the number of errors between baseline and post-test 1, this decrease was not statistically significant. Figures 2–4 illustrate the decreasing trend observed for all these driving errors among baseline, post-test 1, and post-test 2.

## Discussion

The aim of this pilot study was to determine whether the DriveFocus™ app intervention could impact the number of visual scanning, adjustment to stimuli errors, and total driving errors made by young novice drivers, by comparing pre- and post-intervention outcomes.

Our study consisted mostly of female, Caucasian participants. Male young drivers are overrepresented in motor vehicle collisions among youth. For example, in the United States in 2015 alone, the number of road traffic-related fatalities among males between the ages of 16 and 20 years of age doubled that of females (36). Thus, a larger scale trial will require recruitment enhancement strategies to increase the representation of males to assess whether there is a gender-specific impact of the intervention.

Although most participants had a G1 license (59%; first level of Ontario’s graduated licensing program), a close 41% had a G2 license. Thus, the sample had similar representation of youth at the stages of the graduated licensing program. Further analysis will look to discern whether any differences between participants with a G1 or G2 license are present when comparing pre- and post-test outcomes. Also, most participants in the sample received a variation of formal driver’s education, course instruction, and/or on-road training. However, only three participants reported receiving computer-based training and one simulator training. These numbers reflect limited access or availability of such forms of training. However, the evidence indicates limited impact of traditional driver’s education on hazard anticipation skills, and points to the promising effects of computer or multimedia training (17). Much of the participants reported not having been involved in a crash or having received a traffic ticket or citation. However, without access to police reports the information could not be confirmed.

Regarding their driving habits, most participants reported they had never or only occasionally engaged in behaviors such as exceeding the speed limit, answering or making a call while driving, or texting while driving. This information is not consistent with a recent report released by the AAA Foundation for Traffic Safety, indicating that 88% of young drivers had engaged in at least one risky behavior on the wheel in the previous 30 days (37). However, this report also indicates that many young drivers perceive the behavior to be acceptable, which could explain under reporting. A larger sample is required to empirically validate this finding.

Visual scanning, adjustment to stimuli, and total errors significantly decreased from baseline to post-test 2, indicating potential efficacy of the intervention. In addition, adjustment to stimuli errors decreased from post-test 1 to post-test 2. The decrease in the number of errors indicates that participants were better able to detect and respond to roadway hazards. Participants in this pilot study completed the app sessions under supervision. However, the DriveFocus™ app is delivered on an iPad (currently available on iTunes) and could be completed by the young driver at home. The latter provides a cost-effective way of delivering a hazard anticipation intervention that builds on the mobile technology preferences of youth. A follow-up study is currently underway to investigate participants’ perceived acceptability and suitability of this intervention. Finally, capitalizing on the use of dynamic displays, the DriveFocus™ intervention provides an opportunity for the driver to increase their understanding of potential hazard and critical roadway information, and potentially improving their ability to gaze at latent and covert hazards and react in a timely manner. However, a study including a control group (e.g., receiving the RAPT program) is needed to confirm and empirically validate these findings, while overcoming the limitations of the current study.

### Limitations

Given the pilot nature of this study, our findings have limited internal and external validity. First, males were underrepresented in our sample, indicating the need for enhanced recruitment procedures in a larger trial. Second, our findings are not generalizable to young drivers as a population, as our sample consisted exclusively of young drivers (primarily females) licensed in the province of Ontario, Canada. In addition, our analysis includes novice drivers with and without prior driving experience. Third, participants may have exerted extra caution in their post-test as a result of their awareness of the assessment and intervention (i.e., Hawthorne effect). Although the simulator allowed for reproducible and consistent assessment across participants, and the randomization of the three testing drives across participants controlled for the potential learning effect of the drive, our study did not include a control group necessary to control for limited internal validity. Thus, without a between-subjects design, our conclusions regarding the efficacy of the DriveFocus™ intervention are preliminary and findings should be interpreted with caution.

## Conclusion

By empirically testing an intervention aimed at improving teen’s visual scanning and attention to critical road information, and building on teens’ mobile technology preferences, this study provides early support for the preliminary impact of the DriveFocus™ app. Given a statistically significant decrease in the number of visual scanning, adjustment to stimuli and total number of driving errors—after six interventions—our results warrant further studies. Future studies must overcome the limitations of this study as follows: include a control group match for gender, age, and license status; include teens from diverse geographic regions; and control for driving experience to make a more definitive conclusion about the effect of the DriveFocus™ intervention.

## Ethics Statement

This study was approved by the University of Western Ontario, Non-medical Research Ethics Board (#107267). The study was also registered as an intervention trial in ISRCTN (study ID: ISRCTN66950576). All participants provided informed consent prior to enrolling in the study. For participants under 18 years of age, the parent or legal guardian provided informed consent. Participants received a $25 movie theatre gift card and a $20 gas voucher for their participation in the study.

## Author Contributions

LA was responsible for overall study procedures, data collection, data analysis, and manuscript preparation. SC provided study conceptualization, overall guidance in the data collection process, as well as feedback on each round of manuscript drafts. SM was a research assistant on the project and responsible for participant recruitment and data collection. MK was responsible for all simulator evaluations. WH provided assistance and guidance pertaining to methodology and statistical analysis.

## Conflict of Interest Statement

The authors wish to declare that the second author has a professional relationship (previous grants, publications, and presentations) with one of the developers of the app. However, no members of the research team (including all authors and research assistants) have any financial interest in the funding company, the app, or any related products.

## References

[1] World Health Organization. Global Status Report on Road Safety 2015. Geneva, Switzerland (2015). Available from: http://www.who.int/violence_injury_prevention/road_safety_status/2015/en/ (Accessed: February 5, 2018).

[2] National Center for Health Statistics. Mortality among teenagers aged 12–19 years: United States, 1999–2006. NCHS Data Brief (2010) 37:1–8. 20450538

[3] Pradhan AK Hu X-S Buckley L Bingham CR Pre-frontal cortex activity of male drivers in the presence of passengers during simulated driving: an exploratory functional near-infrared spectroscopy (fNIRS) study. Paper Presented at the 8th International Driving Symposium on Human Factors in Driver Assessment, Training, and Vehicle Design Iowa City, IA, USA (2015).

[4] Steinberg L Risk taking in adolescence. Curr Dir Psychol Sci (2007) 16(2):55–9. 10.1111/j.1467-8721.2007.00475.x

[5] McCartt AT Mayhew DR Braitman KA Ferguson SA Simpson HM. Effects of age and experience on young driver crashes: review of recent literature. Traffic Inj Prev (2009) 10(3):209–19. 10.1080/15389580802677807 19452361

[6] O’Malley PM Johnston LD Driving after drug use or alcohol use by American high school seniors, 2001–2011. Am J Public Health (2013) 103(11):2027–34. 10.2105/AJPH.2013.301246 24028266 PMC3828684

[7] Durbin DR McGehee DV Fisher D McCartt A. Special considerations in distracted driving with teens. Ann Adv Automot Med (2014) 58:69–83. 24776228 PMC4001672

[8] National Highway Traffic Safety Administration. Traffic Safety Facts 2015. Washington, DC (2015). Available from: https://crashstats.nhtsa.dot.gov/Api/Public/ViewPublication/812384 (Accessed: February 5, 2018).

[9] Foss RD Williams AF Adolescent drivers: fine-tuning our understanding. J Adolesc Health (2015) 57(1):S1–5. 10.1016/j.jadohealth.2015.04.024 26112733

[10] McDonald CC Curry AE Kandadai V Sommers MS Winston FK Comparison of teen and adult driver crash scenarios in a nationally representative sample of serious crashes. Accid Anal Prev (2014) 72:302–8. 10.1016/j.aap.2014.07.016 25103321

[11] McDonald CC Goodwin AH Pradhan AK Romoser MRE Williams AF. A review of hazard anticipation training programs for young drivers. J Adolesc Health (2015) 57(1 0):S15–23. 10.1016/j.jadohealth.2015.02.013 26112734 PMC4483194

[12] Lee SE Klauer SG Olsen ECB Simons-Morton BG Dingus TA Ramsey DJ Detection of road hazards by novice teen and experienced drivers. Transp Res Rec (2008) 2078:26–32. 10.3141/2078-04 19169380 PMC2630240

[13] Pradhan AK Hammel KR DeRamus R Pollatsek A Noyce DA Fisher DL. Using eye movements to evaluate effects of driver age on risk perception in a driving simulator. Hum Factors (2005) 47(4):840–52. 10.1518/001872005775570961 16553070

[14] Fisher DL Laurie NE Glaser R Connerney K Pollatsek A Duffy SA Use of a fixed-base driving simulator to evaluate the effects of experience and PC-based risk awareness training on drivers’ decisions. Hum Factors (2002) 44(2):287–302. 10.1518/0018720024497853 12452274

[15] Mayhew DR Simpson HM Williams AF Ferguson SA. Effectiveness and role of driver education and training in a graduated licensing system. J Public Health Policy (1998) 19(1):51–67. 10.2307/3343089 9581430

[16] National Highway Traffic Safety Administration. Research Agenda for an Improved Novice Driver Education Program. Washington, DC (1994). Available from: https://ntl.bts.gov/lib/25000/25800/25848/DOT-HS-808-161.pdf (Accessed: February 5, 2018).

[17] Petzoldt T Weiss T Franke T Krems JF Bannert M. Can driver education be improved by computer based training of cognitive skills? Accid Anal Prev (2013) 50:1185–92. 10.1016/j.aap.2012.09.016 23058654

[18] McDonald CC Kandadai V Loeb H Seacrist T Lee Y-C Bonfiglio D Evaluation of a risk awareness perception training program on novice teen driver behavior at left-turn intersections. Transp Res Rec (2015) 2516:15–21. 10.3141/2516-03 26709331 PMC4689436

[19] Taylor TG Masserang KM Pradhan AK Divekar G Samuel S Muttart JW Long term effects of hazard anticipation training on novice drivers measured on the open road. Proc Int Driv Symp Hum Factors Driv Assess Train Veh Des (2011) 2011:187–94. 10.17077/drivingassessment.1396 25285323 PMC4180240

[20] Crundall D Andrews B van Loon E Chapman P. Commentary training improves responsiveness to hazards in a driving simulator. Accid Anal Prev (2010) 42(6):2117–24. 10.1016/j.aap.2010.07.001 20728670

[21] Isler RB Starkey NJ Williamson AR. Video-based road commentary training improves hazard perception of young drivers in a dual task. Accid Anal Prev (2009) 41(3):445–52. 10.1016/j.aap.2008.12.016 19393791

[22] McKenna FP Horswill MS Alexander JL. Does anticipation training affect drivers’ risk taking? J Exp Psychol Appl (2006) 12(1):1–10. 10.1037/1076-898X.12.1.1 16536655

[23] Monahan M A Usability Evaluation of a Driver Training Application for Teens with Autism Spectrum Disorder. (Unpublished Doctoral Project). St. Paul, MN, USA: St. Catherine University (2016).

[24] Connelly LM Pilot studies. Medsurg Nurs (2008) 17(6):411–2. 19248407

[25] Whitehead AL Julious SA Cooper CL Campbell MJ. Estimating the sample size for a pilot randomised trial to minimise the overall trial sample size for the external pilot and main trial for a continuous outcome variable. Stat Methods Med Res (2016) 25(3):1057–73. 10.1177/0962280215588241 26092476 PMC4876429

[26] Classen S Monahan M Brown KE Hernandez S Driving indicators in teens with attention deficit hyperactivity and/or autism disorder. Can J Occup Ther (2013) 80(5):274–83. 10.1177/0008417413501072 24640642

[27] Ali EK El-Badawy SM Shawaly EA Young drivers behavior and its influence on traffic incidents. J Traffic Logist Eng (2014) 2(1):45–51. 10.12720/jtle.2.1.45-51

[28] Reynolds C Comprehensive Trail Making Test (CTMT). Austin, TX: PRO-ED, Inc (2002).

[29] Smith A Symbol Digits Modalities Test. Los Angeles: Western Psychological Services (1982).

[30] Bruininks RH Bruininks BD Bruininks-Oseretsky Test of Motor Proficiency. 2nd ed Minneapolis, MN: Pearson Assessments (2005).

[31] Beery KE Beery NA The Beery-Buktenica Developmental Test of Visual-Motor Integration. 6th ed Bloomington, MN: Pearson (2010).

[32] Brooks JO Goodenough RR Crisler MC Klein ND Alley RL Koon BL Simulator sickness during driving simulation studies. Accid Anal Prev (2010) 42(3):788–96. 10.1016/j.aap.2009.04.013 20380904

[33] Gianaros PJ Muth ER Mordkoff JT Levine ME Stern RM. A questionnaire for the assessment of the multiple dimensions of motion sickness. Aviat Space Environ Med (2001) 72(2):115–9. 11211039 PMC2910410

[34] Classen S Bewernitz M Shechtman O. Driving simulator sickness: an evidence-based review of the literature. Am J Occup Ther (2011) 65:179–88. 10.5014/ajot.2011.000802 21476365

[35] Classen S Levy C Meyer DL Bewernitz M Lanford DN Mann WC. Simulated driving performance of combat veterans with mild tramatic brain injury and posttraumatic stress disorder: a pilot study. Am J Occup Ther (2011) 65(4):419–27. 10.5014/ajot.2011.000893 21834457

[36] National Highway Traffic Safety Administration. Distracted Driving 2013. Washington, DC (2015). Available from: https://www.distraction.gov/downloads/pdfs/Distracted_Driving_2013_Research_note.pdf (Accessed: February 5, 2018).

[37] AAA Foundation for Traffic Safety. 2017 Traffic Safety Culture Index. Washington, DC (2017). Available from: http://aaafoundation.org/wp-content/uploads/2018/03/TSCI-2017-Report.pdf (Accessed: April 24, 2018).

